# The effect of pestle acupuncture for patients with lactation insufficiency after cesarean section: Study protocol for a randomized controlled trial

**DOI:** 10.1097/MD.0000000000023808

**Published:** 2021-01-22

**Authors:** Dongshuang Li, Yunlan Jiang, Xiaoqing Ma, Qing Li, Xin Chu, Wei Zhong, Xiaochun Deng, Xiaolian Yang

**Affiliations:** a Nursing School, Chengdu University of Traditional Chinese Medicine; b Department of Obstetrics; c Department of Operation, Hospital of Chengdu University of Traditional Chinese Medicine, Chengdu, Sichuan Province, PR China.

**Keywords:** cesarean section, lactation insufficient, pestle acupuncture, pestle needle, protocol, randomized controlled trial

## Abstract

**Introduction:**

Cesarean section is a common operation in obstetrics, and the incidence of insufficient breast milk is high in parturients undergoing cesarean section. Studies have shown that acupuncture or massage at related acupoints can promote the secretion and excretion of milk. These external treatments are quick, safe, and effective. On the other hand, they can avoid the potential risk of changes in milk composition that may be caused by the use of drugs. Pestle needle therapy is a new branch of traditional acupuncture, and pestle needle operation does not need to break the skin. The pestle needle has good clinical efficacy and safety in cervical spondylosis, insomnia, fatigue, depression, and so on, but few studies have focused on the effect of pestle acupuncture for patients with lactation insufficiency after cesarean section. This study aims to determine whether pestle needle therapy is effective and safe in the treatment of postpartum milk deficiency.

**Methods:**

This is a 2 parallel-group, assessor-blinded, randomized controlled trial.128 patients with lactation insufficient after cesarean section will be recruited and randomly divided into control group and the pestle needle group in a 1:1 ratio. The control group will receive routine nursing care of milk deficiency. In the pestle needle group, pestle needles will be used to operate on the acupoints such as bilateral Shao ze (S11), bilateral Ru gen (ST18), Dan zhong (DU14), 8 array acupoints of Shen dao (DU11) and so on. It will be operated once a day for 5 days. The primary outcomes are milk yield, degree of breastfilling, degree of milk siltation and other milk deficiency symptom, and serum prolactin. Secondary outcomes include syndrome of traditional Chinese medicine, such as facial expression, fatigue, loss of appetite, and so on.

**Discussion:**

Pestle needle therapy based on acupoint and meridian theory may increase milk secretion and excretion, which will provide a new intervention means to promote breastfeeding and have great significance to guide clinical treatment.

**Trial registration number:**

ChiCTR2000039752.

## 1 Introduction

In some parts of China, the rate of cesarean section is as high as 43.82%,^[1]^ and the rate of advanced age puerpera is as high as 50.34%.^[2]^ Between 2008 and 2013, the rate of cesarean section in southeastern Brazil increased from 35.1% to 42.7%.^[3]^ compared with mothers who had spontaneous vaginal delivery, mothers who had cesarean section delivery were lower in the rate of exclusive breastfeeding and had 2.25 times the odds of giving prelacteal feeds.^[4]^ Traditional Chinese medicine (TCM) believes that fasting before cesarean section, loss of blood during operation and poor diet after operation lead to biochemical passivity of milk caused by deficiency of both qi and blood.^[5]^ On the other hand, incision pain, poor sleep, and depression lead to galactostasis by liver qi stagnation. Modern medicine found that the neutrophil-lymphocyte ratio in the blood of mothers who had cesarean section was higher than that of vaginal delivery, and the synthesis of mitochondrial adenosine triphosphate decreased, resulting in delayed lactation or lactation insufficiency.^[6,7]^ Breastfeeding can provide the most suitable nutrients and adequate water for the child, regulate intestinal flora,^[8]^ prevent infection, reduce neonatal mortality,^[9]^ enhance brain development, reduce postpartum hemorrhage, reduce the risk of breast and ovarian cancer, reduce postpartum pain,^[10]^ promote postpartum recovery, and emotional communication between mothers and infants.^[11]^ The lack of breast milk has corresponding harm to both mother and baby.

**Figure 1:**
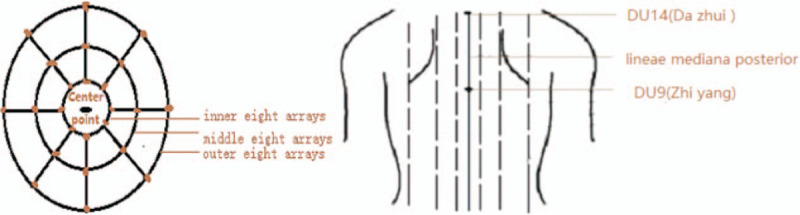
Unique acupoints of pestle acupuncture.

**Figure 2:**
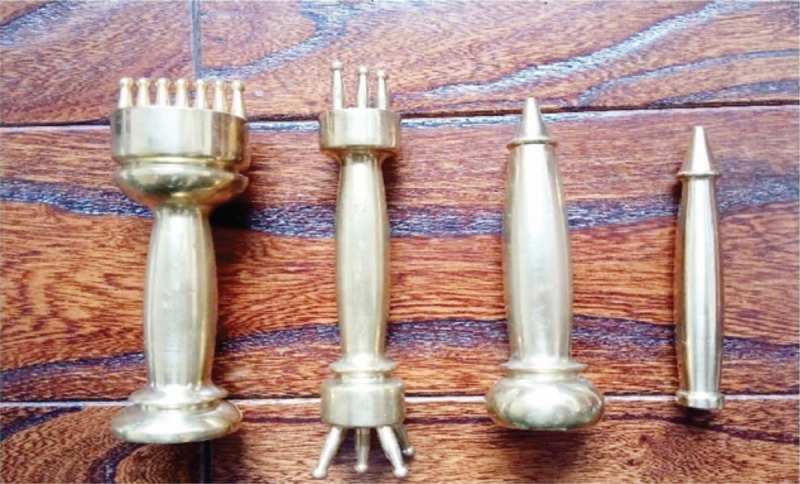
Pestle needle tool (from right to left): Kuixingbi, Jingganchu, Wuxingsantaichu, and Qiyaohunyuanchu.

Pestle acupuncture as a unique branch of traditional acupuncture,^[12]^ its thought of syndrome differentiation is consistent with the theory of TCM, and its characteristic acupoints are Bazheng acupoints (8 array acupoints) and Heche Road (the road for the vehicle).^[13]^ Draw a circle with 1 acupoint as the center and a certain distance as the radius, and then divide the circle into 8 equal parts to form 8 array acupoints in the outer circle. The distance from the center of the circle to the outer 8 array is divided into 3 equal parts, which is drawn into 2 circles that form the inner 8 array and the middle 8 array. The pathway which qi and blood run is called the Heche Road. There are 7 Heche Roads on the back, which one is the posterior median line and the other 6 are parallel lines of 0.5 cun, 1.5 cun, and 3 cun apart from the posterior median line. There are 4 different specifications of pestle needle tools for operation of different acupoints. Kuixingbi is about 8 cm in length, which one end is oval shape and the other end is blunt pestle. Jingganchu is about 10.5 cm in length, which one end is circular arc and the other end is blunt pestle. Wuxingsantaichuis about 11.5 cm in length, which one end is 3 pins in line and the other end is 5pins in blossom shape. Qiyaohunyuanchu is about 10.5 cm in length, which one end is circular arc and the other end is 7 pins stand in parallel. The special acupoints and tools of the pestle needle are shown in Figures 1 and 2.

Compared with traditional acupuncture, pestle needle does not pierce into the skin and avoids the pain that may be caused by acupuncture. At present, The pestle needle has good clinical efficacy and safety in the treatment of more than 10 diseases such as cervical spondylosis, insomnia, lumbocrural pain, sub-health, and so on.^[14–17]^ There are more than 200 articles of pestle needle published on China National Knowledge Infrastructure, but there is no English literature involved. This plan aims to introduce this special school of acupuncture and promote the spread of pestle needle. At the same time, it also explores the effective intervention therapy of pestle for postpartum milk deficiency.

## 2 Methods/Design

### 2.1 Study design

**Figure 3:**
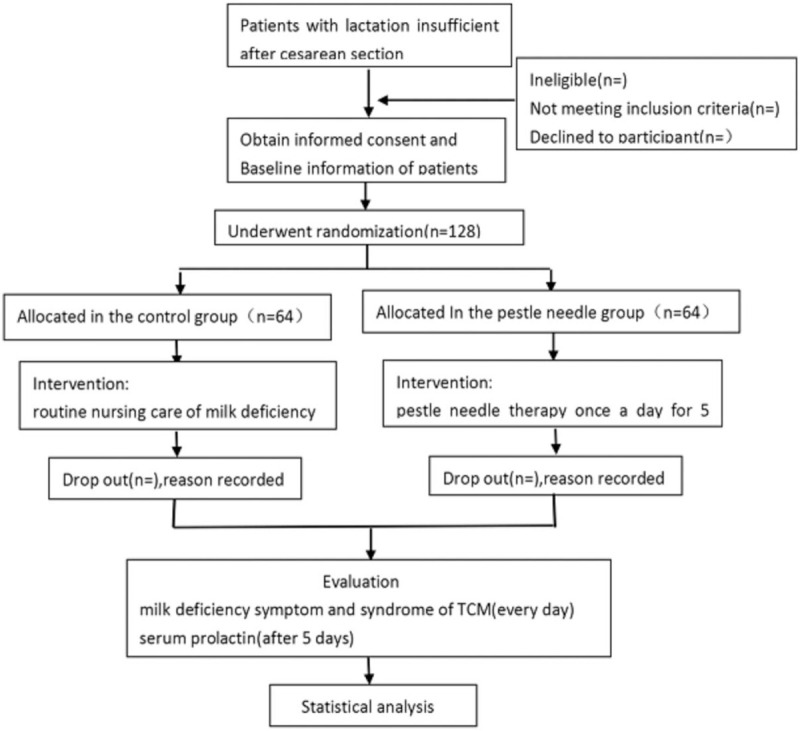
Trial flow chart.

This is a pragmatic, parallel, 2-armed randomized controlled exploratory study. The protocol was reported in accordance with the Standard Protocol Items. The flow chart of the experiment is shown in Figure 3.

### 2.2 Study setting, recruitment and ethics

This study was approved by the Ethics Committee of the affiliated Hospital of Chengdu University of TCM, and will operate in the Department of Obstetrics of the affiliated Hospital of Chengdu University of TCM. The study plans to recruit 128 patients with lactation insufficiency after cesarean section. The researchers will select qualified subjects according to strict inclusion and exclusion criteria, and patiently inform the subjects of the purpose, method, benefits, and risks of the study. Sign a written informed consent form after obtaining the patient's consent. Patient privacy will be protected and each patient will be assigned a unique random number, which is the unique direct identifier on all case report forms.

### 2.3 Inclusion criteria

(1) Diagnosis of lactation insufficiency according to the standards for diagnosis and curative effect of Chinese medical symptom criteria.^[18]^
(2) Women diagnosed as lactation insufficiency in 48 hours after delivery.
(3) Age 20 to 40 years.
(4) Women undergoing cesarean section at 37 to 42 weeks of pregnancy.
(5) The Apgar score of baby is 8 to 10.^[19]^
(6) The length of hospital stay is not less than 7 days to guarantee the completion of 5 treatments.
(7) Willing to breastfeed.
(8) Sign the informed consent form and volunteer to participate in this study.

### 2.4 Exclusion criteria

(1) Do not exhaust or eat after cesarean section.
(2) Abnormal breast development, breast feeding difficulties caused by a history of mastitis, sunken nipples, and chapped nipples.
(3) With serious primary diseases or psychosomatic diseases.
(4) With gestational diabetes mellitus and gestational hypertension.
(5) With postpartum hemorrhage and history of allergy.
(6) Have taken drugs for the treatment of postpartum milk deficiency.
(7) With skin allergy and intolerance to pestle

### 2.5 Randomization and blinding

The randomization sequence will be generated by computer using excel software (Excel 2010 developed by Microsoft; Washington, DC), and using block randomization with a block size of 4 via a central randomization system. Put the random distribution card into opaque envelopes and number the envelopes. The number of the envelopes is the same as that of the random distribution card. When qualified subjects enter the study, open the envelopes in order and carry out treatment according to the grouping. The randomized scheme will be designed by a nursing graduate student in the research group, who will not participate in the clinical implementation phase of the study. Data collectors and statistical analysts will not know the grouping of patients. It is a pity that patients and operators could not be blinded in this study.

### 2.6 Interventions

According to the literature review and pestle acupuncture therapy,^[20,21]^ the following necessary acupoints are selected:bilateral Shao ze (S11), bilateral Ru gen (ST18), Dan zhong (RN 17), 8 array acupoints of Shen dao (DU11) which is formed with Shen dao (DU11) as the center and 3 cun as the radius., Heche Road of Da zhui (DU14) to Zhi yang (DU9). And then, add other acupoints according to different syndrome types of lactation insufficiency. Bilateral Zu san li (ST36) andbilateral Xue hai (SP10) will be used for type of deficiency of qi and blood. Bilateral Tai chong (LR3) and bilateral Qi men (LA14) will be used for type of liver qi stagnation (Table 1). Treatment will be performed with Taiji pestle needle tool which was designed by Li's pestle needle inheritance studio of Chengdu University of TCM. The needle is a patented product and its patent number is 89213016.4. The operator use pestle tool 1 or pestle tool 2 to knock on and stimulate S11, ST18, RN 17, ST 36, SP 10, LR 3, and LA 14 down to the point that the patient can bear, and then relax gently. Next, use pestle tool 3 to stick on the skin and repeatedly draw an S-shaped or 8-shaped figure in 8 array acupoints of Shen dao. Last, The operator use pestle tool 4 massage along the Heche Road of DU14 to DU9. Each treatment lasts 30 minutes, and the strength of the operation should be a slight flush on the skin. Operators need to receive professional training in the operation of pestle needles in advance.

**Table 1:** Locations for acupuncture.

Acupoint	Location
ST18 (Ru gen)	Below the nipple, at the root of the breast, the fifth intercostal space, 4 cun from the anterior median line
ST 36 (Zu san li)	Three cun directly below ST35, and 1 finger-breadth lateral to the anterior border of the tibia
S 11 (Shao ze)	On the ulnar side of the distal segment of the little finger, and 0.1 cun from the nail angle
LR 3 (Tai chong)	On the dorsal side of the foot and in the depression in front of the junction of the first and second metatarsal bones.
DU 9 (Zhi yang)	On the posterior midline, in the depression under the spinous process of the seventh thoracic vertebra.
DU 11 (Shen dao)	On the posterior midline, in the depression under the spinous process of the fifth thoracic vertebra.
DU 14 (Da zhui)	On the posterior midline, in the depression under the spinous process of the seventh cervical vertebra.
RN 17 (Dan zhong)	On the midpoint of the line between the 2 nipples and the intersection of the fourth intercostal space and the anterior midline
SP 10 (Xue hai)	On the medial thigh, 2 cun above the patella, where the medial thigh muscle processes
LA 14 (Qi men)	Below the nipple, the sixth intercostal space, 4 cun from the anterior median line

### 2.7 Control group

Breastfeeding for 8 to 10 times a day to stimulate the continuous secretion of milk. Keep breasts clean and let baby suck 1 breast empty and then suck the other. Pregnant women should eat a balanced diet eat soups in each meal, such as fish soup, chicken soup, pig's foot soup and so on. Encourage patients’ families to communicate more with parturients in order to reduce maternal tension. Maintain pregnant women in a good mood and get enough sleep.

### 2.8 Pestle needle group

On the basis of the control group, pestle needles will be used for treatment group. The operation time is 30 minutes once a day for 5 days.

### 2.9 Primary outcome measurement

The score of milk deficiency symptom and serum prolactin will be taken as the main outcome indicators. The score of milk deficiency symptom include the evaluation of milk yield, degree of breast filling, degree of milk siltation, the quality of milk, the condition of a baby after breastfeeding (Table 2). The total score was between 0 and 28, and the higher the score was, the more serious the milk deficiency symptom was. Among them, the degree of breast filling and the degree of milk siltation should take the average score according to the situation of both sides of the breast.

**Table 2:** The score of milk deficiency symptom.

	Score			
Item	0	2	4	6
Milk yield	Can fully meet the needs of the baby	Can meet 2/3 of the baby's needs	Can meet 1/3 of the baby's needs	There is almost no milk to feed the baby.
Degree of breast filling	The breasts are full, there is a slight swelling pain, and the milk can flow out without squeezing	The breasts are obviously full, and the milk can flow out with squeezing gently	Breast filling is not obvious, and the milk can flow out with squeezing hard	Breast filling is not obvious, and the milk cannot flow out with squeezing hard
Degree of milk siltation	No milk siltation	Have a feeling of milk distension, and cannot relief after breastfeeding.	The breast has tenderness	Persistent pain caused by milk siltation
The quality of milk	Thick	Clear	No milk	
The condition of a baby after breastfeeding	The baby sleeps quietly between 2 breastfeeding.	The baby sleeps a little quieter between 2 breastfeeding.	The baby cries between 2 breastfeeding.	There is almost no milk to feed the baby.

### 2.10 Secondary outcome measurements

The score of TCM symptoms was taken as the secondary outcome indicator, including lusterless complexion, fatigue, loss of appetite, pain in chest and flank, depression, and body fever (Table 3). According to the situation, each TCM symptom is divided into none, mild, moderate, severe, and the corresponding scores are 0, 2, 4, and 6.

**Table 3:** The score of traditional Chinese medicine symptoms.

	Score			
Item	0	2	4	6
Lusterless complexion	No	Mild: a slight pallor in the face.	Moderate: lips are pale, but not reach the level of anemia.	Severe: lips are obviously pale and reach the level of anemia.
Fatigue	No	Mild: feel fatigue after heavy activity.	Modera: feel fatigue after moderate activity.	Severe: feel fatigue after mild activity.
Loss of appetite	No	Mild: find food tasteless, no reduction in food intake.	Moderate: A loss of appetite and a slight reduction in food intake.	Severe: have no appetite and hardly eat.
Pain in chest and flank	No	Mild: happens occasionally and can be relieved by itself.	Moderate: happens frequently and does not affect the work.	Severe: happens Continuous and affect the work.
Depression	No	Mild: depression in a small part of the time.	Moderate: depression in most of the time.	Severe: depression in all the time.
Body fever	No	Mild: The body is slightly hot, without sweating.	Moderate: The body is hot and slightly sweaty.	Severe: The body is hot and sweating continuously.

### 2.11 Data collection and management

The evaluation of the curative effect and data collection will be carried out by members of the research group except the operator. The symptoms of milk deficiency and TCM symptoms will be evaluated when the patients are enrolled in the group and after each treatment. It will be recorded 6 times. In addition, subject's serum prolactin levels should be measured at the time of entering the group and after the end of whole treatment. First, the evaluator will use the paper version of the evaluation form to record data, and then enter the data into excel form for management. The original case report form and all other forms (including consent forms) will be archived securely in the obstetrics department of Chengdu University of TCM.

### 2.12 Sample size

The formula of comparison of the mean of 2 samples in completely random design was adopted to calculate the sample size:n1 = n2 = 2[(Zα/2+Zβ) σ /δ]2. We set α at 0.05 (2-sided), β at 0.1, and after looking up the table we get Z α /2 at 1.96, Z β at 1.28. In this study, milk yield was selected as an index to estimate sample size. After consulting the reference,^[22]^ we can get σ at 1.23, δ at 0.77, After calculation, the sample size of each group should be around 53. Considering a dropout rate of 20%, a total of 128 participants will be included in this trial.

### 2.13 Statistical analysis

We will use SPSS 21.0 software (SPSS Company, Chicago, IL) for statistical analysis. The measurement data will be expressed by mean ±standard deviation. *T* test will be used for measurement data that obey normal distribution, otherwise Mann–Whitney *U* test will be used for analysis. Analysis Of variance will be used In comparing the changes of lactation deficiency symptom and TCM syndrome in different time between test group and control groups. The counting data will be statistically described by frequency, constituent ratio, percentage and so on, and analyzed by Chi-squared test. All the tests in this paper will be 2-sided, and *P* < .05 will be considered statistically significant.

### 2.14 Ethics and dissemination

The subject was approved by the Research Ethics Committee of the affiliated Hospital of Chengdu University of TCM (NO.2020KL-037). At the same time, it has been registered on ClinicalTrials.gov (NoChiCTR2000039752) and will be reported in accordance with the CONSORT statement. The results of the pilot study will be published in peer-reviewed journals.

## 3 Discussion

Acupuncture and massage are important interventions for the treatment of postpartum milk deficiency and have a good effect.^[21,23,24]^ Some research statistics show that DU 14, ST 18, S 11, ST 36, LR 3 are the acupoints used to treat milk deficiency for the most times.^[25,26]^ In this study, classical effective acupoints will be used, which reflects the scientificity of clinical research. At the same time, 8 array acupoints and Heche Road will be used, which reflects the innovation of clinical research. Eight array acupoints are based on traditional acupoints and integrate the thoughts of the Book of Changes to form special acupoints and arrangement.^[27]^ Using the pestle needles to stimulate the Heche Road in the back can dredge patient's vital qi smooth and make them feel warm, relaxed, and comfortable.^[28,29]^ Through the carding of the meridians and collaterals, it can promote pleasure, relieve pain, eliminate tension, and indirectly promote milk secretion and excretion.

Pestle needle therapy is based on the theory of Tibetan image and meridian in TCM, and the thought of syndrome differentiation in the process of treatment is the same as that of TCM. The pestle needle tool is similar to 1 of the ancient 9 needles recorded in the classic of TCM.^[12,30]^ In a word, pestle needle has the same origin as traditional acupuncture and belongs to a new branch of traditional acupuncture therapy. Furthermore, it has the following advantages compared with traditional acupuncture. Firstly, it is a non-invasive and painless operation, it provides a new treatment scheme for patients with dizzy acupuncture or those who reject traditional acupuncture. Secondly, the operation of pestle needle is relatively easy to learn and master, which makes it easier to promote and apply. Thirdly, the pestle needles combined with related techniques can have the effect of massage.

This study also has some limitations. Due to the particularity of the intervention, this study is unable to blind the operators and subjects, which may affect the results in theory.

## Acknowledgments

We are thankful to the Affiliated Hospital of Chengdu University of Traditional Chinese Medicine for funding this study.

## Author contributions

**Conceptualization:** Dongshuang Li, Qing Li, Wei Zhong.

**Funding acquisition:** Yunlan Jiang.

**Investigation:** Qing Li, Wei Zhong, Xiaochun Deng, Xiaolian Yang.

**Supervision:** Yunlan Jiang, Xiaoqing Ma, Xin Chu.

**Writing – original draft:** Dongshuang Li.

**Writing – review & editing:** Yunlan Jiang.
